# Supplementary material for: Structure and Assembly of the Proteus mirabilis Flagellar Motor by Cryo-Electron Tomography
Source: Int J Mol Sci. 2023 May 5;24(9):8292. doi: 10.3390/ijms24098292 (PMC10179241; doi:10.3390/ijms24098292)
Supplement: Supplementary file 1 [file ijms-24-08292-s001.zip › ijms-2337003 movie legend.pdf]

Movie S1: A cryo-electron tomogram of a vegetative P. mirabilis cell illustrating the presence of many fimbriae stemming from the cell. Scale bar is 100 nm.

Movie S2: A cryo-electron tomogram of a swarmer P. mirabilis cell illustrating the presence of many peritrichous flagella stemming from the cell. Scale bar is 100 nm.
