# Supplementary material for: Structure and Assembly of the Proteus mirabilis Flagellar Motor by Cryo-Electron Tomography
Source: Int J Mol Sci. 2023 May 5;24(9):8292. doi: 10.3390/ijms24098292 (PMC10179241; doi:10.3390/ijms24098292)
Supplement: Supplementary file 1 [file ijms-24-08292-s001.zip › ijms-2337003-figure.pdf]

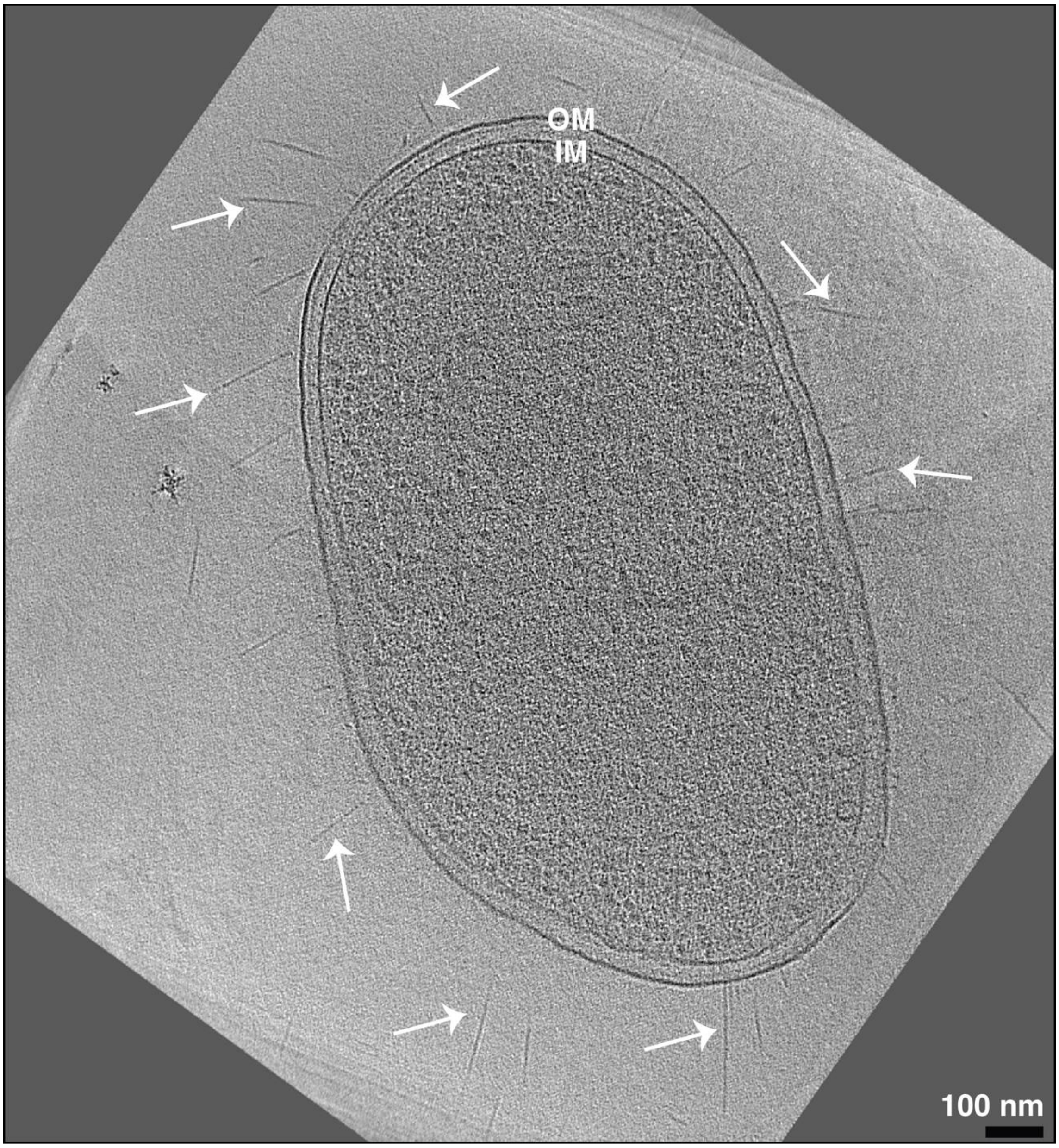

Figure S1: A slice through a cryo-electron tomogram of a *P. mirabilis* vegetative cell indicating the presence of multiple fimbriae stemming from the cell (white arrows). Scale bar is 100 nm.

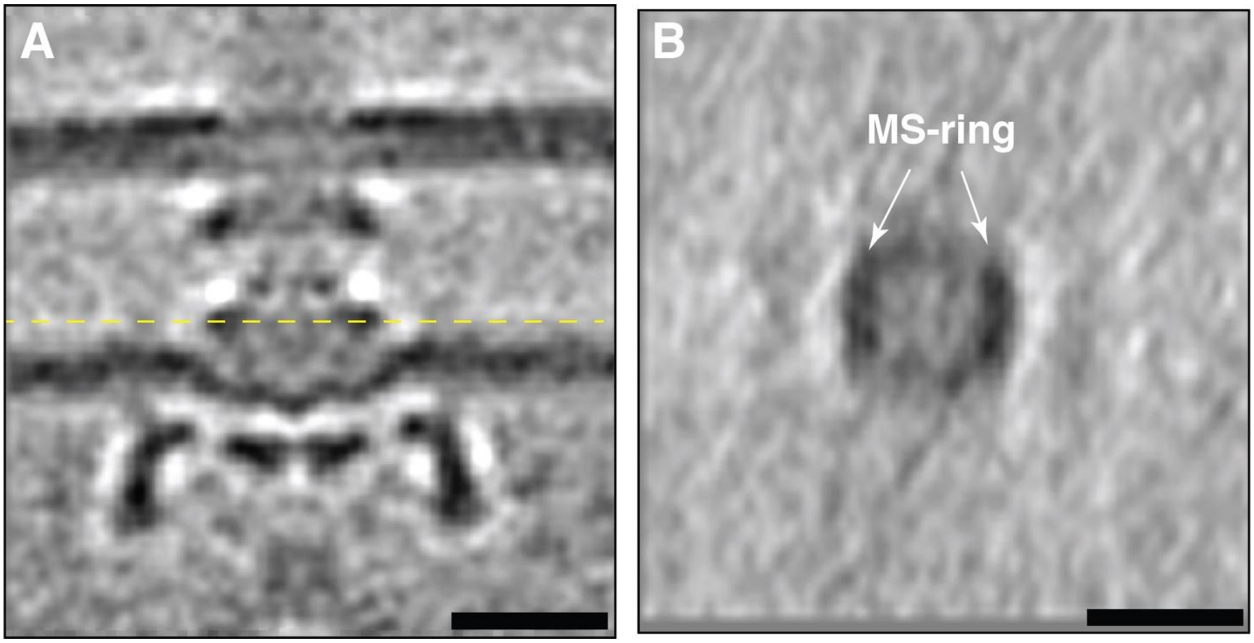

Figure S2: A) A slice through the subtomogram average of *P. mirabilis* flagellar motor with a cross section through the motor (indicated by the dotted yellow line) shown on the left in panel (B). Scale bar is 20 nm.
